# 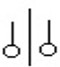 Side by Side: Reflections on Two Lifetimes of Dance

**DOI:** 10.3389/fpsyg.2021.587379

**Published:** 2021-03-12

**Authors:** Ann Kipling Brown, Anne Penniston Gray

**Affiliations:** ^1^Arts Education Program Faculty of Education, University of Regina, Regina, SK, Canada; ^2^Saskatchewan Association of Social Workers, Regina, SK, Canada

**Keywords:** dance, education, embodiment, access, participation, self-care

## Abstract

Telling stories about our experiences in dance brings to light unconscious knowledge and memories of the past and helps us understand our own decisions and practices. Reflexivity and story telling is central in the process of remembering and embodies some of the key aspects of autoethnography as a research tool. We are directed to examine and reflect on our experiences, analyzing goals and intentions, making connections between happenings and recounting each single experience. Dance has the potential for positive impact on both physical and mental health among professional dancers as well as among dance students and has the power to connect them to culture and community in unique and important ways. Research has provided evidence that arts engagement provides positive forms of social inclusion, opportunities to share arts, culture, language, and values and points to the value of the arts in the prevention and amelioration of health problems. Together with those benefits of a dance experience there is clear evidence of what can be learned in, through and about dance. In this time of the Covid-19 pandemic it seemed more relevant and poignant to examine our own experiences in dance as well as those experiences of others that have influenced our lives.

## Introduction

Kipling Brown: I am in my senior years and have had a rewarding and fulfilling career in dance, specifically dance education where I have worked with children and youth in studio and educational settings, and teacher training in college and University programs. It has been an interesting process to reconnect with those experiences, tedious at times and cathartic at others as I recounted and explored my various dance experiences. Many were so vivid and clear while others had faded and jumped out as I tracked a time when I danced or taught or choreographed. I wrote in my journal as I decided to retire from my University teaching position: Dance will always be present in my life even though I may not move as my inner dancer believes (June 10, 2013). I was astonished at the many activities I recalled and that so many had brought joy and a sense of accomplishment while others had produced stress and doubt in my abilities.

Penniston Gray: I am over 60 which shocks me as I type this as in my head I am about 35 but as I add up the things I have done in my life I am conscious of the contributions I have made to my profession and my community. I am a therapist in private practice and have been a therapist for over 30 years aside from a break to work for the Faculty of Social Work at the local University. For 20 years of that time I worked exclusively with women who experienced gender based violence. It is heavy work and while my practice is broader than it was when I worked exclusively with women who had experienced interpersonal violence and abuse I recognize it still takes a toll on my heart. I am thinking about how I might slow down a bit but so far have not been very successful, as I love my work. Dance has been a part of my self-care strategy in some way for most of those 30 years. I am still dancing and have been enjoying belly dance classes over the past 3 years and dance using YouTube videos in my home office.

## Our Connection

We have been connected for 25 or so years. We first danced together on Monday afternoons in a class offered by the University conservatory program during the mid nineties. Penniston Gray wrote, I think I was the youngest person in that class and I was so encouraged by the other dancers, as I felt at home and safe to stretch myself in that environment. Dancing together in this way led naturally to me being one of the participants in Kipling Brown's Thursday contemporary class, this too was a safe space in which to explore moving. Both classes offered me respite from the stressors of the day and were an integral part of my self-care plan for at that time I was very busy and spent a great deal of my time and energy in my head and dance required that I inhabit my body. Kipling Brown was teaching in the Arts Education Program at the University of Regina at that time and wanted to take a class, which she was not leading. She wrote, I really wanted to dance and the class offered some structured sequences as well as some improvisation. I had such fun being a student and dancing with other like-minded individuals. I was reconnected to my passion for my own dancing, feeling good in my body and spirit. It was at this time that I wrote about the power of dance and also choreographed an autobiographical solo that expressed my identity and the intersectionality of my identities as a woman who dances, educates, researches and advocates.

This experience led to subsequent dance connections between the authors, one of which was co-facilitated workshops for women who desired resources and space to renew, create, express, heal, and connect to other women. Together with class participation the authors also choreographed and performed many pieces over the years, for example a composition for an International Women's Day celebration as well as a composition that was part of a conference presentation.

## Our Process and Methodology

This project stemmed from an interest in what dance has meant to us and how those interactions with others in dance have had a profound affect on our lives. To explore our experience in dance and to add our voices to the literature on dance and dance education we chose to use autoethnography, a method that would provide a means of analyzing evidence and systematic reflection and present a record and empower discovery. Autoethnography is seen as both process and product and has come to be accepted as an acceptable platform for the practice of research. Ellis et al. (2011) describe autoethnography as a combination of “characteristics of autobiography and ethnography” and that “writing autobiography, an author retroactively and selectively writes about past experiences.” The authors identify that through comparing and contrasting personal experience against existing research new perspectives or “epiphanies” may emerge (Ellis et al., 2011). Autoethnography facilitates a more personal point of view by emphasizing reflexivity and personal voice and as Russell posits such personal testimonials/stories “assumes a site of authenticity and veracity (Russell, 1999).” Autoethnographies would involve the retelling and reinterpretation of our personal histories and as such ethical issues of objectivity and legitimacy are of concern. Thus, the highly personalized process generated some anxiety as we reflected on how our experiences and ideas would be judged. However, we were encouraged by the strong wish of qualitative researchers to discover views of others and accept and value multiple viewpoints.

We began our stories by meeting to discuss our collaborations as students, teachers, and dancers. These dialogues were rich in memories and thoughts about our experiences and connections in and through dance. We referenced our dance involvements by referring to our various journals and writings and found searching our journals and writing for this paper both therapeutic and enlightening. Firstly, we decided to work on our individual timeline of dance involvements both individually and with one another. Secondly, we continued to meet and identify connections between happenings and recounts of those personal experiences, identifying values, themes, issues that came up from our reflective writing and memories. Thirdly, we investigated research and writings that supported the findings of our own experiences and those of other dance participants and scholars. And, finally we have narrated our findings, sometimes together and sometimes individually, in this paper.

## Today

In the midst of our process and journey down memory lane the Covid-19 pandemic arrived and communities were forced into self–isolation. This seclusion provided much time to reminisce and reflect and to continue dancing alone and later virtually; however, the consequence of this time spent alone did not always have the desired outcome. We both found we missed the spontaneity in life and opportunities to meet family and friends and to attend dance classes. And then there was the change in where we could work and meet and the never-ending cancellation of classes, conferences, performances, and meetings. At first, the virtual substitute for these interactions could not replace the meaningful and fulfilling role of those activities in our lives.

The politicians and media talked about the new normal and we contemplated what that would mean to us. Dance companies, schools, and studios provided access to classes and performances, which, at first, compensated for those in person classes. Penniston Gray wrote, Interestingly enough in these strange times in the late winter and spring of 2020 I have found an adult ballet barre class on YouTube. While I am spending my time at home in the evening after a day of virtual counseling sessions I go back to my dance roots for the familiar and comforting structure of ballet barre, for it doesn't require a great deal of space during a time when we are at home social distancing. While I am dancing at home alone I realize my enjoyment of dance is not just about the dance itself, it is about my group of kindred spirits. I am missing dancing with other women as we are socially isolating. In my journal, I wrote, it is a powerful thing that transcends the technique, skills and music. It is about emotion and connection and history (December 13, 2019). In 1997 I wrote of the role of dance in my life and my concerns about how I would fill my time post Bachelor of Social Work (BSW), I'm so glad that I dance though—it is a definite source of pleasure—pure and simple, not driven by clocks or calendars, and a way of breaking down barriers (March 24, 1997). I look forward to dancing with my women again enjoying the steps, the movement but even more, the sisterhood.

For Kipling Brown it was the viewing of Anne Marie De Keersmaeker's choreography, Rain, with music by Steve Reich and made available by Sadler's Wells (2020) that prompted the following journal entry. I was captivated by the pure dance she is known for with the mathematical patterns, mesmerizing variations and geometric use of the stage. The performance lifted my spirits and made me realize that this was a new phase in experiencing and sharing dance with others (April 12, 2020). In a later journal entry, Dance has shown resilience in responding to the affects of the COVID-19 pandemic that is damaging many lives and livelihoods. I have been invigorated by the creativity and generosity of dance artists and companies as they have found ways to share their work virtually (April 2, 2020).

We considered that maybe there would be changes as a result of the fallout from COVID-19 in how we lived our lives and contemplated what changes there would be for dance. We are living in a time of uncertainty and would that redefine what dance is and its importance in our lives? Physical distancing is so unusual for dance artists and participants. The art form that usually depends on coming together has been moved to an online environment; however, not all performances or events have been able to move online. Would people return to dance in their studios or perform works on the traditional stage? Or would people prefer to enjoy dancing and viewing dance virtually? Would some of those benefits of dance that we talk about be lost? Maybe choreographers would take fewer risks on new and challenging work, and producers will provide light-hearted, commercially driven shows to help profits. In light of these questions it seemed even more pertinent to consider what dance means for us. We both recognize that we have been involved in dance for many reasons and that some experiences have provided therapeutic benefits for us and that dance can lead to social cohesion and inclusion for those vulnerable and excluded groups. We, like many others, came to dance in varying ways and found a way from those beginnings to make dance a forceful part of our lives. There are many reasons why people participate in arts activities including arts experiences as a child, parental role models, financial resources, socioeconomic status, degree of participation in leisure activities, and education in the arts.

## The Role of Dance in People'S Lives

Kipling Brown: My passion for dance was always with me but my interest in dance education began during my final years at Grammar School. I was quite athletic, excelling in games and gymnastics and participating in many teams and competitions. Our young and forward thinking Physical Education teacher introduced my class to modern educational dance and gymnastics, firstly, I believe, wanting to introduce more modern approaches, and secondly believing that it would engage more students in physical activity. At this time she also arranged for a group of us to attend a workshop organized by the Manchester Dance Circle. We traveled by public transport to a secondary school on the outskirts of Sheffield and spent the afternoon in a dance session led by Lisa Ullmann. The session was transformative for me, a profound sense of expression and freedom in dance that I had never felt before. It was a time in Britain when dance as a means of education of the whole person was supported and taught in many training colleges and secondary schools. I was inspired by these revelations about what dance could be and applied and was accepted to attend a College of Education where the focus was on Laban's principles of movement. After teaching for a few years I wanted to go further with those studies and so I attended the Art of Movement Studio in Addlestone, Surrey. From 1966 to 1979 I worked in the British state education system where modern educational dance, then renamed creative dance, was integrated in the school curriculum for primary and secondary students. My teaching, choreography, and research in the University setting have continued to reflect Laban's educational and pedagogical practices as well as his principles in choreutics, effort analysis and notation (September, 2008).

Rudolf Laban offered a new rationale for the study of movement and dance for theater and education, stating that we need to go beyond the study of “each particular movement, the principle of moving must be understood and practiced” (Laban, 1948, p. 10) and that the experience can lead to an understanding of self in relation to others. The foundations of his work stems from Ausdruckstanz, a theater dance practice which he pioneered with Mary Wigman in the early twentieth century. The dance form challenged the industrialization and urbanization of modern life. Many dance artists became involved in the practices and principles of Ausdruckstanz to consider the education of all in dance as well as the professional dancer's education (Preston-Dunlop, 1998, p. 165). From those early developments and the subsequent work of many of his followers Laban's influence can be found in many related areas where movement is integral, such as drama education, movement and dance therapy, non-verbal communication, choreology, and cultural studies.

In his seminal writing, Modern Education Dance, Laban (1948, p. 11) expressed that his approach to the study of dance and movement was appropriate to dance education because of “the beneficial effect of the creative activity of dancing upon the personality of the pupil.” His creative and educational approaches were fundamentally the practice of critical pedagogy as discussed by Heiland (2016) “that what we now call ‘reflexivity' a tenet of critical pedagogy, which Laban described as a ‘thought round,' in which dance is understood and experienced through the body, mind, and spirit.” To summarize the influential work of leaders in the field (Freire, 1972; Giroux, 1994; Apple and Au, 2009), the objectives of critical pedagogy are to make education more culturally relevant, inclusive, and accessible to students and through reflexivity and the discussion of teaching and learning students are empowered to address issues that are of interest and relevance to their lives. In dance education this process of reflexivity has been encouraged; for instance, Stinson identified a process for pre-school children as “interaction” defined as “a series of connected and related responses between the children and the teacher (Stinson, 1988, p. 72).” Burridge and Svendler Nielsen (2018) state that creative and empowering pedagogies are important while responding to diversity of needs, the specific role of dance in varying contexts and cultural differences.

Laban's belief that dance should be made available to everyone was reflected in many school curricula and organizations particularly where child-centered education was valued (Thornton, 1971). Many viewed that understanding oneself and one's relationship with others in the world was the most important purpose of dance. A significant contribution to this debate came from the Dance and the Child Conference, held in 1978 in Edmonton, Alberta. At that conference Hill's important message in her keynote presentation was that creative dance experienced by the child as a performer, creator, and spectator would help to develop aesthetic perception, to learn a language to express ideas and imagery and to critically examine dance (Hill, 1978, p. 64). The impact of this conference shaped the organization, Dance, and the Child International (daCi) where Laban's foundational principles are reflected in the presentations of dance educators, scholars and young dancers.

Nevertheless, beginning in the 1970s these goals were disrupted by a shift of emphasis on dance, particularly in high schools and college/University programs, to a focus on dance as a body of knowledge to be learned rather than as a means to individual growth. Despite this change many educational and professional dance contexts continued to recognize the benefits of engagement in dance for the learner and the development of self. Bond and Stinson (2001, p. 4) investigated the role of dance in young people's lives and what it means to them. They found that the children talked about the importance of dance and identified “states of being that are in some way significantly different from the everyday.” Conner et al. (2020, p. 3), found that “culturally oriented dance has strong ties to health benefits and stress reduction for participants of varying ages and backgrounds.” Supporting such health benefits Snow (2019) identifies that strategies in managing emotions and coping with stress are developed. Additionally the benefits to learning are identified by Thomas et al. (2015) who indicate that perseverance, attention, motivation and self-confidence in the arts transfer to other subjects and people's lives. Website: SI News^1^ supports this transfer of skills describing that higher grades in mathematics and reading are achieved through arts activity. McClanahan and Hartmann (2018) describe that leadership, artistic and teamwork skills are developed and Kiseda and Bowen (2019) affirm that there are less disciplinary infractions. Davis (2017) in advocating for arts education emphasizes the need for institutions and governments to pay attention to the importance of creativity and the way it promotes problem solving, inventiveness, social innovation and entrepreneurship, as well as communication and expression. Finally a study conducted by Kipling Brown et al. (2015) participants summarized the above-mentioned values of a dance education. The participants responded to questions about their experiences, describing how dance was their way of living in the world and that it was good not only for their physical but social and emotional well-being. Many believed it was important to the development of a cultural sense as well as an aesthetic sense. In conclusion, it is imperative that we offer all students a dance education that not only fosters an understanding of dance and its many manifestations but also offers a means to live an active and meaningful life.

Penniston Gray: I had always wanted to dance as a child but I either didn't communicate that well enough to my parents or they didn't have resources to send me to dance. I was not an athletic child and by my recollection I was quite uncoordinated, I played softball and would pray that the ball didn't come my way as I feared being hit by the ball. I dreamed of being a ballerina and graceful but I was very conscious that I had a weight problem and felt uncomfortable in my body. I do remember enjoying the dance part of gym class and vividly remember learning the Virginia Reel and the Bunny Hop, neither of which I have ever danced again. I think I was about 10 when we learned the Virginia Reel and it was so strange to have to dance with even more uncomfortable sweaty handed boys. My thoughts at this stage in life are that if more dance had been offered in school that would have allowed me to quench and build on my desires to dance. Reflecting on this in light of the above discussion of the many ways that dance and creativity foster growth and self development makes me feel sadder that that little girl didn't have that as her creative language then.

## How do People Access Dance?

Many people's experiences in dance, particularly for girls, begin in infancy and continue until teen years and concentrate on training girls in deportment and behavior. It was accessible to those who could afford to pay for lessons and as Clegg et al. (2019) outline “Dance, especially ballet, is traditionally considered a female activity, with a widespread perception that it can affect a child's expressed femininity.” For those who were able to pursue a professional career there were clear expectations regarding the ideal ballerina figure, thus maintaining a specific cultural focus where access is limited. Dance for boys is experienced differently. They continue to be a minority in dance programs as well as in the professional dance world. However, there are exceptions and we see many examples of those who began dancing later in life and continue to dance for recreational purposes or enter professional programs and finally companies or a related dance field. Rodney Diverlus, a prominent and successful dancer, activist, and choreographer today, did not have easy access to dance. He states:

Growing up in a working class immigrant family, I did not have the privilege of attending the ballet, could not afford theater or dance camp, was not able to participate in after-school arts programs, and was not able to afford the drop-in community classes (Diverlus, 2013).

Kipling Brown had her first experience with dance in Primary School. In her journal she wrote of fond memories and that “getting it right” was very important and classmates relying on her to remember the steps and patterns was rewarding. We would walk in twos from our classroom to the hall situated in a beautiful sandstone building across the road from the school. It was the only place with a hall where we could spread out and perform the country-dances that our classroom teacher taught. I remember London Bridge is Falling Down, a round dance game for any number of dancers, going under the arches and taking turns. My best memory is of the longways sets, such as The Black Nag and Brighton Camp. The steps were simple, walking, running, galloping, and skipping, and I loved the intricate patterns of the characteristic formations for partners and groups.

I was 11 years old when I participated in dance at a studio in India, attending classes in ballet, tap and acrobatics with great enthusiasm. At the same time the school provided classes in Manipuri dance with a young professional dancer. I have written and talked about these experiences many times, how they impacted me and changed my ideas and feelings about dance. In particular, I have told the story about my ballet classes. I was becoming very bored with ballet. It did not seem to be dancing at all but a series of exercises that said very little to me about dance and expression. I wanted to discontinue and maybe just take tap and acrobatics, but that was not possible. I had made a commitment and so I continued. Then our ballet teacher left and a new one took her place. This new teacher was older, maybe 50, was tall and elegant and smelled of perfume and cigarettes. She began the class as usual, barre and then center work and some fun traveling. Then she asked if we knew the story of the Dying Swan (which we did). She reviewed it with us and played the music and asked us how we would dance. She asked us to dance our own version. I was in heaven, transported in my story of the dying swan. I loved ballet. I was inspired and believe I understood implicitly why the class was organized in the way it was. I waited every class for that time to improvise (July, 1999). At the same time I enjoyed my Manipuri dance classes. They were joyous and energetic dances and reminded me of the circle dances I had enjoyed in Primary School in the UK. The mudras and facial expressions were enchanting and challenging. A highlight was when we were asked to perform at a Gala event. We had beautiful Manipuri costumes and had our hands and eyes painted. At this time dance was a refuge and comfort for me (July 20, 1999).

Penniston Gray: I came to dance in my thirties and having that maturity allowed me to see dance as something I did for myself, something that was fun and not competitive. I would have loved to dance as a child, in fact I yearned to but that didn't happen. In retrospect that was probably a good thing as my joy in dancing might have been squashed by exams, competitions and costumes that made me uncomfortable. In my thirties, I had a great sense of self and I guess I instinctively knew what would work on my body and what would not. The same lack of coordination identified in my childhood also affected my dance abilities but I was able to take them in stride.

I started with an adult jazz class at the dance school that my then husband worked at as an accompanist for ballet classes. I had no idea where to start taking classes and this was a known entity. When that class finished in the spring I found an adult ballet class, tried that, and loved it. I found another adult ballet class at a different school and continued taking ballet for several years and eventually as my confidence in my abilities (and body) grew I felt I needed more. I needed something that was less steeped in tradition, less rigid, and tried a couple of different contemporary dance classes and loved them. I loved the setting that had more room for creativity and individuality.

We, like many others, came to dance in varying ways and found a way from those beginnings to make dance a forceful part of our lives. Various studies and reports identify why people participate in the arts; for instance, the report, Demographic Patterns in Canadians' Arts Participation in 2016, stated “nearly nine in 10 Canadians attended an arts activity in 2016.” A wide range of demographic factors on participation rates, such as education, family income, language, sex, and age were taken into account to reveal that “[o]ne-half of Canadians 15 or older (50%) made or performed art in some way in 2016, 9% in dance.” Involvement included “art galleries (39%), arts performances or festivals (68%), and movie theaters (71%)” (Hills Strategies Research, 2019, p. 38). Further detailed information about dance across Canada is outlined in Dancing Across the Land: A Report of the Dance Mapping Inventory (2016) funded by Canada Council for the Arts and Ontario Arts Council. This report endeavors to capture all dance genres in all contexts, including professional, recreational and educational settings. The report is extensive and, while it may not at present reveal all that is happening in dance, it does provide information of a high level of participation in dance in Canada at the avocational and professional levels (Canada Council of the Arts, 2016).

Kipling Brown: During my studies and teaching career I have had opportunities to develop my skills, perform, and create and, most of all to mature as a teacher. My philosophy as a dance educator changed and grew to reflect that dance can have particular and different meanings in people's lives and that exploring and creating in dance can have significant impact on thoughts and feelings. My pedagogy reflected the importance of inclusion and diversity and finding ways to provide valuable dance activities for all, young and old. As well as providing structured segments in technique and improvisation together with opportunities to create and perform I implemented bodily writings and reflections. Bodily writings suggested by Foster (1995, p. 3) became an integral part of my practice, encouraging participants to talk about and write about those bodily experiences that effectively “traces the physical fact of movement and also an array of references to conceptual entities and events.” Like Cooper (2011, p. 53) I am particularly interested in how the creative and compositional process in dance making and writing are complementary and that implementing practices of internal reflection on one's own and other's movement and body, drawings, journal writing and discussion lead to an embodied knowing and learning.

At various times I have been asked and encouraged to create experiences for women. One such experience came about from a dance program for children in which each month I would invite the parents in to watch and dance with their children. I could see that many parents were eager to dance and enjoyed moving with their children and when a group of mothers approached me and asked if there could be classes for them I agreed. I initiated my first adult class in 1977 and since that time I have continued to offer a class wherever I have been teaching. It is in one of those classes for women that Penniston Gray and I had chance to work together.

The women's dance classes also inspired me to initiate a series of research studies and formal and informal conversations with women to explore what dance might mean to women who yearn to return to dance or take dance for the first time in their adult lives. Each group of women comprising of women aged 20–60, with varying dance experience, participated in a series of classes designed to explore dance and then reflect on those experiences in conversation and writing. Many compared these classes to previous dance classes commenting on “rigid structure and repetitive nature of previous dance classes”. They wrote about the “passion” and “spirituality” of the dance experiences, that they felt “an increasing sense of freedom” and that it became “exhilarating.” Many expressed discomfort they had trying to connect with the body and rid themselves of the need “to look and feel and act a certain way.” They found that dance helped them to come to terms with problems with body image and the fear of exposing themselves through movement. Many described the friendships made, that they felt a sense of community and were liberated by the joy of moving. These experiences led to more classes and many friendships. For me it was a sense of accomplishment, writing in my journal it felt good to have opened doors into dance for these friends and I am also clearer about the structure and flexibility of the dance class and that to spend more time on dance making is rewarding (April 4, 2006).

Penniston Gray: During my 30's I was busy, I worked full time, was a single parent and was a student working on an undergraduate degree and then a graduate degree all the while balancing those roles. Dance for me was an opportunity to live in my body given that both my career and academics required that I spent the majority of my time in an intellectual place. I recall a time in dance class where I felt as though I was moving from my head into my body, landing in my body with a “thud.” I also remember during the time when I took time off work to write my thesis that it was occasionally challenging for me to make that shift from intellect to body and there would be times that it took too long for my liking to be present in my body and move. These dance classes were a necessary part of my self-care practice, they were something that I did for myself and they required I move my body.

I shared my thoughts regarding the power of dance with clients, some of whom joined dance themselves. I shared also with one of my professors in the Faculty of Social Work in my choice of the topic for directed-studies. The power of dance in my life was most evident in my process journal that documented my experiences in dance class, other movement classes and workshops co-facilitated by the co-author of this article and myself.

Those workshops were an opportunity for me to test my ideas about the healing powers of dance and creativity especially for women and especially for women whose bodies had been violated and betrayed in some way. These workshops, comprising of movement, writing and visual art activities, were offered on Sunday mornings free of charge to ensure accessibility for those who might not have the resources to pay for such experiences. The workshops were also opportunities for me to do my own work around my body. In my journal after one of these Sundays I wrote about the work we had been doing on transitions between levels, “there is a similar feeling at the highest and lowest levels—these places feel much more stable both physically and emotionally, but the transitional phase between them feels less balanced (both physically and emotionally). You're not where you've come from, not yet where you're going—in a place that is unfamiliar, uncomfortable, and one you sense won't be in long enough to get comfortable in. To a degree this parallels the healing process, moving through all sorts of things with some sense of where you want to be, the old places/ways aren't comfortable anymore but those new places you're working through certainly aren't comfortable so you just have to keep going (March 23, 1997).”

The women's dance class where I spent most of my time offered a setting that was not competitive, one that fostered camaraderie and fun and created connection between us all. These women formed my participant pool for my research for Masters in Social Work (MSW) and the act of interviewing them, transcribing those interviews and using their words as the data for my MSW research allowed me to get to know them at a deeper level beyond dancing together. When I interviewed these women one of the questions I explored with them was relating to their identity as a dancer. Most women did call themselves dancers but the most important aspect of this dance class for them was the idea that we were a community and the connection they shared with one another. I will forever be grateful to them for assisting me in achieving my goal (Penniston, 2002).

When we were dancing together, several of us were mums of teenagers and now some of us are grandmothers. We have maintained a friendship for over 25 years with dance as the thread that brought us together.

Connection is also evident in the dance class I am doing now; I am doing Belly Dance with several other women in the teacher's basement. In this class too, there is a range of ages from the teacher's daughter to her mother-in-law. We took a hiatus between Christmas and March and it was so good to get back together as this group too, is not just about dance, it is about a group of people who care about one another. I missed the women as much as the dance.

As a participant, I have been thinking about the similarities between both classes. Both groups of dancers have the commonality that dance brought us together, we love to dance, it calls to us. Both groups have leaders who share power, are encouraging and create a safe space to explore movement and our bodies. While neither instructor is a dance/movement therapist, Loman (2005, p. 68) states that one of the primary goals of Dance Movement Therapy (DMT) is “removing the obstacles people have in expressing themselves, relating to others or accepting their bodies or selves.” As a participant I would have to attest that this has very much been part of the process for me in both groups of women. Therapy is not the intent of either group but I know that in the act of moving I have been moved in an emotional sense. Loman (2005, p. 87) says that DMT “imparts a sense of connectedness and joy” and this is evident in both groups of women as well. Laughter rings out in both groups and there is a sense at least from my perspective that “I have found my people.” In my journal, I noted, Dance is a powerful tool and it is especially powerful as a shared experience with a group of kindred spirits (1997). Fast-forward two decades later I wrote in my journal, I think there is something so powerful about women dancing together. Lorran reminded me that in Belly Dance it is always women dancing with women, they don't dance with men. It's the same type of thing that happened when we women danced together in Kipling Brown's class (December 10, 2019). It is so interesting that my view hasn't changed with the passage of time or with a new group of women.

Self-care is considered by writers Monk (2011) and Blackmon and Hardy (2020) to be an aspect of a Social Worker's professional ethical practice as being impaired by the impact of hearing our clients' stories impacts our ability to be effective helpers. There are many ways to practice self-care and as a therapist I will assist clients in planning their self-care strategies but for me, during the time of COVID, setting and maintaining boundaries is probably most important. Along the way (in a source lost to the passage of time) I heard self-care referred to as being a way to hold space for one's self. That description of it resonates with me for if I don't take care of myself as a helper I am of no use to anyone, least of all to myself. I think a self-care practice is as unique as the individual but it would make sense that effective self-care would address the mental, physical, spiritual, and emotional aspects of the self and dance ticks all the boxes for me. As someone who has challenges with coordination I have to really think about the movement, sort it out in my head before my body can “get it.” The practice of dance is spiritual to me as it moves my emotions and offers space for expression of my spirit and I addressed this in my journal so long ago, Dance provides a safe place in which to express emotion. It can be contained in the movement, released with so much more intensity than one can do in ordinary life. I love this aspect of dance; it has been healing for me (January 27, 1997). A week later I write that dance offers a sense of integration between my body and my head that allows input from my soul (January 3, 1997). My own experience is echoed by Duberg et al. (2016). In their research with adolescent girls with “internalizing problems,” they found that “giving voice to different mental states increases the physical connection with emotions and helps with dealing with the challenges in daily life by identifying, recognizing and affirming different kinds of emotions (Duberg et al., 2016, p. 14).”

## Final Thoughts

Our personal stories have revealed for us the power of dance to heal and nurture our minds, our bodies and our spirits, to provide a space to be creative and express ideas and feelings, and to assist in learning about the world and others. Through our own experiences and the experiences of others we have come to realize the importance of making dance accessible and providing opportunities for all to participate in dance as performers, spectators, and creators. We believe the selected writings of dance scholars, research studies and statistics have supported our journey, identifying the significance of dance in professional, educational and recreational contexts. Our writings came at a time of crisis as the COVID-19 pandemic affected the creative productivity and opportunities of many dance teachers, artists and arts organizations.

Penniston Gray: As the restrictions and concerns about working with individuals in person continue (as I write this I am in week 6 or so of working virtually from home) I have noticed that good intentions I had early on have gone by the wayside, I am not going for walks or doing YouTube barre classes and I feel tired and drained carrying the weight not only of these times but the weight of what I hear from my clients and the weight of the grief my friends are dealing with. I know creative activities such as journaling and playing around with music and movement helps me do the work I do. Self compassion, as identified by Neff (2003), means I am gentle with myself, acknowledging these are strange times and navigating this world means we don't always get it right but we can try to do better. Articles by Baron Cadloff (2020) and Schwartz and Pines (2020) refer to the impact of the “allostatic load” related to the COVID crisis and the ever present stress as we manage in isolation and having very usual things such as grocery shopping become strange with shortages of cleaning products, toilet paper and yeast, one way aisles, and plexiglass shields. Friends experiencing grief cannot be hugged; we maintain a safe distance of 6 feet maybe also wearing masks. These daily activities feel surreal. Making time for activities that are normal and grounding can offer rhythm to the days that don't have the usual rhythm and pace.

This time of COVID with its uncertainty and the necessity of staying apart in order to pull together has brought out the worst in people in the form of hoarding and profiteering but that is dwarfed by a focus on the best in people. I am aware that people are taking care of one another by dropping off meals to family and essential workers, putting hearts and words of encouragement in windows, making noise at prearranged times to thank essential workers and dancing in the streets at an particular time of day. This resilience comes from what we social workers call a “strengths based approach” in that focusing on the strengths and the positive is much more beneficial than focusing on the negative. A client of mine says she is focusing on spending time with her kids that she typically doesn't get, as they aren't at school or at their activities. She described to me that she now realizes that she was exhausted from the busy life she has and this has been a time of rest for her. I have maintained connection with friends and family albeit virtually but a side effect of this time is that one of my friends groups will have a group member attend gatherings virtually, we wouldn't have thought of that before. People have shown ingenuity by using social media to offer creative displays of dance alone or with the magic of technology. A search for the words “dancing during social distancing” on YouTube video on May 5, 2020 yielded over 30 videos. It seems that other people have been using dancing together apart as a means of connecting and supporting. Humans strive toward health and wholeness and are creative and resilient, these strange times have demonstrated that there are threads that weave us together and dance is one of them.

Kipling Brown: Delving into those past memories and writings has provided some solace in this time of crisis. It has been cathartic to reflect upon the many interactions with dancers, artists, and educators I have had through dance. I see this relationship of dancing, teaching and creating as a dialectical process, a place of discourse to clarify the relationship of dance in my life. My life is dance, dance my life. Dance my self-expression, my self expressed in and through dance. My body as dancing, dancing my body. Dance as my cultural expression, my cultural life expressed in dance. Dance as pedagogy, pedagogy as dance (October 19, 1994). Our history and the power of movement are rooted in our bodies. We can come to know others and ourselves through those expressions and, even though in this time of uncertainty we are challenged about the role of dance we know that we will continue to communicate through dance. I am reminded that everyone finds his or her own way in dance, that no one way is the right or only way. In this time of crisis the dance community is persistent and I am excited by how people have engaged in dance and with others, how professional dancers, schools, and companies are generously sharing their teaching and resources and how many have found dance to be a new place to connect with family, friends, and strangers.

## Author Contributions

Both authors listed have made a substantial, direct and intellectual contribution to the work, and approved it for publication.

## Conflict of Interest

The authors declare that the research was conducted in the absence of any commercial or financial relationships that could be construed as a potential conflict of interest.

## Footnotes

^1^Website: SI News—“Can an Arts Education in School Improve Academic Performance?” March 2019 by Study International Research URL: https://www.studyinternational.com/news/can-an-arts-education-in-school-improve-academic-performance/ (Accessed March 22, 2020).
